# Pulse oximetry test for screening congenital heart diseases: a systematic review

**DOI:** 10.1590/1980-220X-REEUSP-2023-0215en

**Published:** 2024-03-01

**Authors:** Gabrielle Freitas Saganski, Márcia Helena de Souza Freire, Wendel Mombaque dos Santos

**Affiliations:** 1 Universidade Federal do Paraná, Departamento de Enfermagem, Curitiba, PR, Brazil.; 2 Instituto Joanna Briggs. São Paulo, SP, Brazil.

**Keywords:** Oximetry, Infant, Newborn, Heart Defects, Congenital, Pediatrics, Systematic Review, Oximetría, Recién Nacido, Cardiopatías Congénitas, Pediatría, Revisión Sistemática, Oximetria, Recém-Nascido, Cardiopatias Congênitas, Pediatria, Revisão Sistemática

## Abstract

**Objective:**

To determine the accuracy of the Pulse Oximetry Test (POT) in screening for Congenital Heart Diseases (CHD) in newborns in the first 48 hours of life.

**Method:**

Systematic review of diagnostic test accuracy with meta-analysis. The selection of studies was carried out in June 2021. Studies were selected with newborns, in a hospital or home environment, without a previous diagnosis of CHD, regardless of gestational age at birth, who underwent POT within the first 48 hours after birth. Registration on the PROSPERO platform – CRD42021256286.

**Results:**

Twenty-nine studies were included, totaling a population of 388,491 newborns. POT demonstrated sensitivity of 47% (95% CI: 43% to 50%) and specificity of 98% (95% CI: 98% to 98%). Subgroup analyses were carried out according to the different testing period, inclusion of retests in protocols and population of premature newborns.

**Conclusion:**

POT is a test with moderate sensitivity and high specificity. It is more effective when carried out within 24h – 48h of birth; in protocols that present retests, within two hours after the first measurement. It does not show satisfactory effectiveness for premature newborns.

## RESUMO

**Objetivo:**

Determinar a acurácia do Teste de Oximetria de Pulso (TOP) na triagem de Cardiopatias Congênitas (CC) em recém-nascidos nas primeiras 48 horas de vida.

**Método:**

Revisão sistemática de acurácia de teste diagnóstico com metanálise. A seleção dos estudos foi realizada em junho de 2021. Foram selecionados estudos com recém-nascidos, em ambiente hospitalar ou domiciliar, sem o diagnóstico prévio de CC, independentemente da idade gestacional ao nascimento, que realizaram o TOP entre as primeiras 48h após o nascimento. Registro na plataforma PROSPERO – CRD42021256286.

**Resultados:**

Foram incluídos 29 estudos, somando uma população total de 388.491 recém-nascidos. O TOP demonstrou sensibilidade de 47% (IC 95%: 43% a 50%) e especificidade de 98% (IC 95%: 98% a 98%). Foram realizadas análises dos subgrupos conforme período de realização do teste diferente, inclusão de retestes nos protocolos e população de recém-nascidos prematuros.

**Conclusão:**

O TOP é um teste de moderada sensibilidade e alta especificidade. Apresenta maior efetividade quando realizado no intervalo entre 24h – 48h do nascimento; em protocolos que apresentem retestes, em até duas horas após a primeira medida. Não apresenta efetividade satisfatória para recém-nascidos prematuros.

## RESUMEN

**Objetivo:**

Determinar la precisión de la Prueba de Oximetría de Pulso (POT) en el screening de Cardiopatías Congénitas (CC) en recién nacidos en las primeras 48 horas de vida.

**Método:**

Revisión sistemática de la precisión de las pruebas diagnósticas con metanálisis. La selección de estudios se realizó en junio de 2021. Se seleccionaron estudios con recién nacidos, en ambiente hospitalario o domiciliario, sin diagnóstico previo de CC, independientemente de la edad gestacional al nacer, a quienes se les realizó POT dentro de las primeras 48 horas después del nacimiento. Registro en la plataforma PROSPERO – CRD42021256286.

**Resultados:**

Se incluyeron 29 estudios, totalizando una población de 388.491 recién nacidos. POT demostró una sensibilidad del 47% (IC del 95%: 43% al 50%) y una especificidad del 98% (IC del 95%: 98% al 98%). Se realizaron análisis de subgrupos según los diferentes períodos de prueba, la inclusión de repruebas en los protocolos y la población de recién nacidos prematuros.

**Conclusión:**

POT es una prueba con sensibilidad moderada y especificidad alta. Es más eficaz cuando se realiza entre las 24 y 48 horas siguientes al nacimiento; en protocolos que presenten repruebas, dentro de las dos horas posteriores a la primera medición. No muestra una eficacia satisfactoria para los recién nacidos prematuros

## INTRODUCTION

Congenital heart diseases (CHD) are defined as abnormalities in cardiocirculatory structure or function during embryonic development. CHDs affect 0.8% of all live births and correspond to the second leading cause of death in children under five years of age. For every ten children with CHD, six are diagnosed late, which causes significant morbidity and mortality. Factors such as gestational age at birth, maternal age, and newborn weight also impact the survival rate^(1,2)^. From 1990 to 2017 there was a 4.2% increase in the prevalence of births with CHD^(3)^.

Globally, 12 million people live with CHD, resulting in approximately 600,000 years lived with disability, according to a recent global report. CHD is among the seven main causes of child death and 2nd among middle and high-income countries^(4)^. Approximately 25% of deaths from congenital anomalies are caused by CHD^(3)^. In 2017, CHD caused around 260,000 deaths, of which almost 70% (180,000 deaths) were of children under one year of age^(4)^.

The literature indicates that diagnoses made during prenatal care have increased, allowing birth planning in a specialized reference center^(5)^. However, the birth of a newborn and discharge from hospital without a diagnosis of CHD is still common, with a rate of 30%. Thus, the POT issue for screening critical congenital heart diseases, which require surgical treatment in the first year of life, is relevant, given the importance of diagnosis before hospital discharge for morbidity and mortality outcomes, patient and family quality of life^(5,6)^.

It is also considered that even though it is implemented in the health system of some countries, including Brazil, challenges are still identified for its implementation and interpretation at a global level, such as the (un)preparedness of the health system, with a focus on infrastructure and in human resources trained to deal with positive cases; there are also divergences regarding recommendations, a fact that affects false-positive results^(5,6)^. Thus, CHDs remain a priority for public health actions.

The Brazilian reality shows that although Neonatal Screening is legally required, according to the guidelines of the Ministry of Health^(7)^, and consists of carrying out five tests in the first 48 hours of the newborn’s life (Pulse Oximetry, Red Reflex Test, Hearing Screening, Tongue Test, and Heel Prick Test), full coverage is not achieved in the ideal period. According to a Brazilian capital cutout, 36.6% of the sample had access to the five recommended tests. Among the tests, the Heel Prick Test, the Red Reflex and Heart Test (Pulse Oximetry) reached more than 90% of the studied population, considering the first 28 days of life^(8)^.

The promotion of Neonatal Screening with POT is considered a strategy to potentially increase CHD detection at birth, for care and for epidemiological surveillance^(4)^. Despite the great advances in the subject of Neonatal Screening, such as the expansion of identified diseases^(9)^ and the incorporation of the specific database for the program^(10)^, improvements are still needed regarding health policies, aiming at comprehensive, safe, quality and cost-effective care, considering, for this purpose, that the Brazilian health scenario has a universal health system, the Brazilian Public Health System, called *Sistema Único de Saúde* (SUS).

Current debates are still necessary regarding the POT flowchart, especially the period during which the test is carried out (before 24 hours or after), considering the hospital discharge of the postpartum woman and newborn, and the interval between additional measures, in suspected cases. The POT protocol in force in Brazil since 2013 is carried out on newborns over 34 weeks, before hospital discharge, between 24 and 48 hours of life, with a retest every 1 hour if the first oximetry indicates a value <95% or a difference that is equal to or higher than 3% between the measurement of the right upper limb and the lower limb. If the altered measurement persists during retesting, the newborn should be referred for an echocardiogram^(6)^.

A recent study presented two significant changes to the POT flowchart, removing the greater than 3% difference between the upper and lower limbs, and performing only one additional measurement in an interval of one hour, instead of two, which implies a simplification in test interpretation^(11)^. On the other hand, a new national guideline changes to two additional measures at intervals of one hour each, justifying a reduction in the number of false positive cases^(6)^.

The bibliographical survey prior to the development of this research is highlighted, on the PROSPERO platform, in the Cochrane Database of Systematic Reviews and JBI Evidence Synthesis databases, and identified studies that are similar to this review^(12, 13, 14)^. However, this systematic review was maintained because it is different, given the four-year interval, considering the most recent publication and the inclusion of seven studies after that date, in addition to the inclusion of studies different from the other reviews. Furthermore, it differentiates itself by developing POT analysis in premature newborns and in the situation of deliveries/births at home, as suggested by a previous publication^(12)^. A meta-analysis was carried out in a subgroup according to the period in which the POT was carried out and the number of retests after the first measurement, whose outcomes were added by the researchers, in response to the need presented by recent publications^(6,11)^. For this reason, it is not described in the protocol for this review.

In this context, knowing the correct parameters and possible test changes guarantees better screening. This review aims to equip health professionals working in hospital and home contexts to make decisions about the use of POT aiming at the timely detection of CHD. The objective of this study is to determine the accuracy of the Pulse Oximetry Test to screen for congenital heart diseases in newborns in the first 48 hours of life.

## METHOD

This is a systematic review of diagnostic test accuracy carried out following the JBI^(15)^ and PRISM *extension for Diagnostic Test Accuracy* (DTA) *Studies* ^(16)^ recommendations. The Protocol is registered on the PROSPERO platform under the number CRD42021256286 and published in a journal^(17)^.

The systematic review of diagnostic test accuracy uses the PIRD mnemonic to construct the research question, where P – population, I – Index test, R – Reference test, D – diagnosis of interest^(15)^. This review had the following research question: “What is the diagnostic accuracy of pulse oximetry for neonatal screening of congenital heart diseases in newborns?”

### Data Sources and Research Strategy

The search was carried out in June 2021 using a search strategy developed with the support of a professional librarian, using indexed descriptors and keywords. The following MESH descriptors related to population, diagnosis of interest and index test were used: infant; newborn; neonate; neonatal; premature; preterm; oximetry; pulse oximetry; pulse oximetry screening; heart defects; congenital; abnormality; heart; congenital heart defects; malformation of heart; congenital heart disease; and, malformation. These were associated with Boolean operators (OR and AND) to develop the strategies (Chart S1).

Databases searched included: Cumulative Index to Nursing and Allied Health Literature (CINAHL) and *Excerpta Medica Database* (Embase) and search portals PUBMED, Scopus and *Web of Science,* and the bases for unpublished studies, such as Catalog of Theses and Dissertations of the Coordination for the Improvement of Higher Education Personnel (CAPES) – Brazil, Open Access Theses & Dissertations (OATD) and WorldWideScience.org. The search in the respective databases was carried out through registration with the Federated Academic Community (CAPES CAFe).

### Inclusion and Exclusion Criteria

Studies that had newborns up to 28 days of age as participants were included, regardless of gestational age at birth; as a diagnostic test, the Pulse Oximetry Test; diagnosis of interest, severe congenital heart disease; and as outcomes, sensitivity and specificity data. Studies published in English, Spanish, and Portuguese were considered for inclusion in this review, without time limits. Review studies, letters to the reader, editorials, comments, abstracts of papers presented at events and expert opinions are excluded.

### Study Selection

All citation records from the search were loaded into Endnote and duplicates were removed. Two reviewers independently read the titles and abstracts for evaluation according to the inclusion criteria for the review. No disagreements were identified at this stage. Subsequently, the full text was read independently to qualify the studies. According to inclusion disagreements regarding 176 articles, a third reviewer with experience in systematic reviews by JBI was invited.

### Data Extraction

To describe the articles, the following information was collected, attributed to the study: doi, year of publication, country of authors, authors, title, language, journal, method, approach, general objective, main findings, and recommendations. About the diagnostic test: screening protocol, comparator, context, incidence, false positive, false negative, true positive, true negative, sensitivity, specificity, positive predictive value, false positive rate, negative predictive value. To carry out the meta-analysis, the false positive, false negative, true positive, true negative data were analyzed with the aid of the software *MetaDisc* 1.4^(18)^.

### Assessment of Methodological Quality

The assessment of the methodological quality of the studies was carried out using JBI standardized *check list* for diagnostic accuracy studies^(15)^. To interpret this assessment, the authors categorized three possible levels of methodological quality (high, moderate, or low). If the study presented more than seven positive responses, it was classified as having high methodological quality; if it presented between six and four positive responses, it was classified as having moderate methodological quality; and if it presented between three and zero, it was classified as having low methodological quality.

The referred check list consists of four groups of questions about participant inclusion, index test, reference test, flow and time. The questions are: Was it a consecutive or random sample of enrolled patients? Was a case-control design avoided? Did the study avoid inappropriate exclusions? Were the index test results interpreted without knowledge of the reference standard results? If a retention was used, was it pre-specified? Is the reference standard likely to correctly classify the target condition? Are reference standard results interpreted without knowledge of index test results? Was there an appropriate gap between the index text and the reference standard? Did all patients receive the same reference standard? Were all patients included in the analysis?

### Assessment of the Quality of the Outcome

It used the system *Grading of Recommendations, Assessment, Development and Evaluation* ^(19)^ (GRADE) through the GRADEpro platform to evaluate the strength of recommendation and quality of evidence for the outcomes of diagnostic accuracy studies at one of four levels (high, moderate, low, and very low), according to the study design, indirect evidence, inconsistency, and publication bias.

### Data Synthesis

To carry out the meta-analysis, false positive, false negative, true positive and true negative data were analyzed with the support of MetaDisc 1.4 software. Meta-DiSc is a precise and comprehensive meta-analysis software for diagnostic test accuracy studies. All computational algorithms in it have been validated through comparison with different published statistical tools and meta-analyses.

### Ethical Aspects

There was no need for approval from the Research Ethics Committee, as it was a review study.

## RESULTS

**Figure 1:**
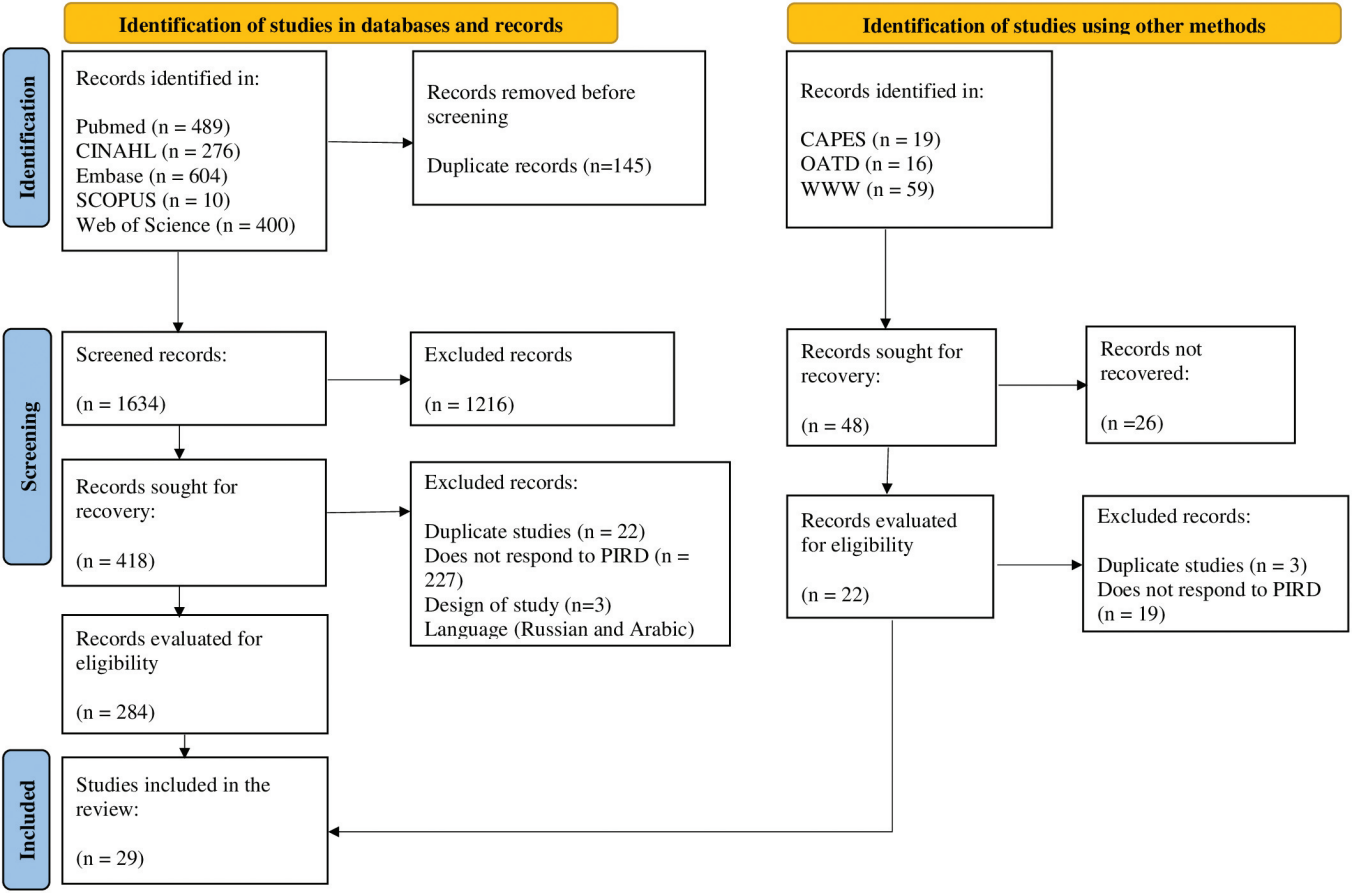
Flowchart of the process of identification, screening, and inclusion of systematic review articles. Curitiba, PR, Brazil, 2023. Source: The author (2023). Legend: CAPES – Coordination for the Improvement of Higher Education Personnel; CINAHL – Cumulative Index to Nursing and Allied Health Literature; OATD – Open Access Theses & Dissertations; PIRD – Population, Test Index, test Reference and Diagnosis of interest; WOS – Web of Science; WWW – World Wide Science.

The search resulted in 1873 studies identified in databases and gray literature. A total of 145 duplicates were excluded and, in the study identification tab using other methods, which included gray literature, 26 were not recovered as the file was not available in full. After the eligibility process, 29^(20–48)^ studies, published between 2002 and 2021 remained. The description of the study selection phases, including selection, inclusion, and justification of excluded studies, is presented in Figure 1. Analysis of the methodological quality of the included studies demonstrated that all were classified as having high methodological quality, as they met the 10 evaluation criteria, according to the JBI standardized checklist for diagnostic accuracy studies.

The total population of the included studies consisted of 388,491 newborns. The predominant language in which the studies were published was English and the type of study was observational, retrospective and prospective. Nine studies^(20–22,25,29,30,33,39,43,44)^ followed a protocol to perform the test in the first 24 hours, and the others from 24 hours after birth to 48 hours.

There was a plurality in terms of study countries, demonstrating the divergences in the protocols followed, with the United States^(21,23,25,28,32,35,47)^ and United Kingdom^(30,40–43)^ being the countries with the most publications. As for the sample, it varied in the size of the studies. Most of them analyzed one hospital, but there were studies analyzing more than 14 hospitals^(23,33–44)^. Chart 1 demonstrates the characteristics of the studies, considering participants, test period, cutoff points, and care behaviors.

**Chart 1:** Characteristics of the included studies. Curitiba, PR, Brazil, 2023.

Author and year	N	PRT	Oxygen saturation cut-off point and care procedures
Almawazini et al.^(20)^	2,961	21 to < 40	≥95%: no additional procedures. 90–94%: new assessment in 1 hour. If lower than 95% persisted, an echocardiogram was performed. <90%: echocardiogram performed.
Andrews et al.^(21)^	1,908	24 to 48	≥95%: no additional procedures. <95%: three new assessments in 1, 2 and 3 hours. If lower than 95% persisted, an echocardiogram was performed. <90%: echocardiogram performed.
Arlettaz et al.^(22)^	3,257	6 to 12	≥95%: no additional procedures. 90–94%: new assessment in 6 hours. If lower than 95% persisted, an echocardiogram was performed. <90%: echocardiogram performed.
Van Naarden Braun et al.^(23)^	3,423	24 to 48	≥95%: no additional procedures. <95%: two new assessments in 1 and 2 hours. If lower than 95% persisted, an echocardiogram was performed. <90%: Echocardiogram was performed.
Cubells et al.^(24)^	8,856	24 to 48	≥95%: no additional procedures. 90–95%: new assessment in one hour. If lower than 94% persisted, an echocardiogram was performed. <90%: admission to the NICU and echocardiogram.
Diller et al.^(25)^	77,154	up to 24	≥95%: no additional procedures. 90–94%: new assessment between 1 and 2 hours. If lower than 94% persisted, an echocardiogram was performed. <90%: echocardiogram performed.
Donia and Tolba^(26)^	120	2 to 24	≥95%: no additional procedures. <95%: new assessment in 2 hours. If lower than 95% persisted, an echocardiogram was performed.
Gamhewage et al.^(27)^	8,718	24 to 48	≥95%: no additional procedures. <95%: echocardiogram performed.
Gong et al.^(28)^	11,322	24 to 48	≥95%: no additional procedures. 90–94%: new assessment between 1 and 2 hours. If lower than 94% persisted, an echocardiogram was performed. <90%: echocardiogram performed.
Havelund et al.^(29)^	2,796	up to 24	≥95%: no additional procedures. <95%: two new assessments in 30 minutes and 1 hour. Pediatric evaluation and echocardiogram persisting below 95%.
Jones et al.^(30)^	10,260	2 to 24	≥95%: no additional procedures. <95%: new assessment in 2 hours. If lower than 95% persisted, admission to the NICU and an echocardiogram was performed. <90%: admission to the NICU and echocardiogram.
Kardasevic et al.^(31)^	1,745	24 to 48	≥95%: no additional procedures. <95%: new assessment in 1 and 2 hours. If lower than 95% persisted, an echocardiogram was performed. <90% echocardiogram performed.
Manja et al.^(32)^	1,445	24 to 48	≥95%: no additional procedures. <95%: new assessment in 1 and 2 hours. If lower than 95% persisted, an echocardiogram was performed. <90%: echocardiogram performed.
Meberg et al.^(33)^	48,686	up to 21	≥95%: no additional procedures. <95%: clinical examination or echocardiogram.
Meberg et al.^(34)^	57,909	24 to 48	≥95%: no additional procedures. <95% were symptomatic and referred for evaluation by a pediatrician. Asymptomatic patient re-evaluated within 3 hours and persisting <95% the pediatrician was called.
Miller et al.^(35)^	1,600	24 to 48	≥95%: no additional procedures. <95%: new assessment in 1 and 2 hours. If lower than 95% persisted, an echocardiogram was performed. <90%: echocardiogram performed.
Mohsin et al.^(36)^	1,650	24 to 48	≥95%: no additional procedures. ≤94%: new evaluation in one hour. If lower than 94% persisted, an echocardiogram was performed.
Mosayebi et al.^(37)^	413	24 to 48	≥95%: no additional procedures. <95%: new assessment in up to 2 hours. If lower than 95% persisted, an echocardiogram was performed. <90%: echocardiogram performed.
Özalkaya et al.^(38)^	8,208	24 to 48	≥95%: no additional procedures. <95%: echocardiogram performed.
Patriciu et al.^(39)^	5,406	up to 24	≥95%: no additional procedures. <95%: echocardiogram performed.
Prudhoe et al.^(40)^	29,930	2 to 48	≥95%: no additional procedures. <95%: echocardiogram performed.
Richmond et al.^(41)^	5,626	24 to 48	≥95%: no additional procedures. <95%: new assessment in up to 2 hours. If lower than 95% persisted, an echocardiogram was performed.
Saxena et al.^(42)^	19,009	up to 48	≥95%: no additional procedures. <95%: echocardiogram performed.
Singh et al.^(43)^	25,859	up to 12	≥95%: no additional procedures. <95%: new assessment in up to 2 hours. If lower than 95% persisted, admission to the NICU and an echocardiogram was performed.
Tautz et al.^(44)^	3,336	6 to 36	≥95%: no additional procedures. 90–94%: new assessment in 4 and 6 hours. If lower than 95% persisted, an echocardiogram was performed. <90%: echocardiogram performed.
Tsao et al.^(45)^	6,296	24 to 36	≥95%: no additional procedures. <95%: three new reviews in 30 minutes, 1 hour and 1 hour and 30 minutes. Persisting below 95% echocardiogram performance.
Vaidyanathan et al.^(46)^	5,487	up to 48	≥95%: no additional procedures. ≤94%: echocardiogram performed.
Walsh^(47)^	14,564	24 to 48	≥94%: no additional procedures. <94%: assessment by a pediatric cardiologist.
Zayachnikova et al.^(48)^	20,547	24 to 48	≥95%: no additional procedures. <95%: echocardiogram performed.

Source: The author (2023). Legend: ID – Identification of the article. N – number of study participants. PRT – Test completion period in hours.

**Figure 2:**
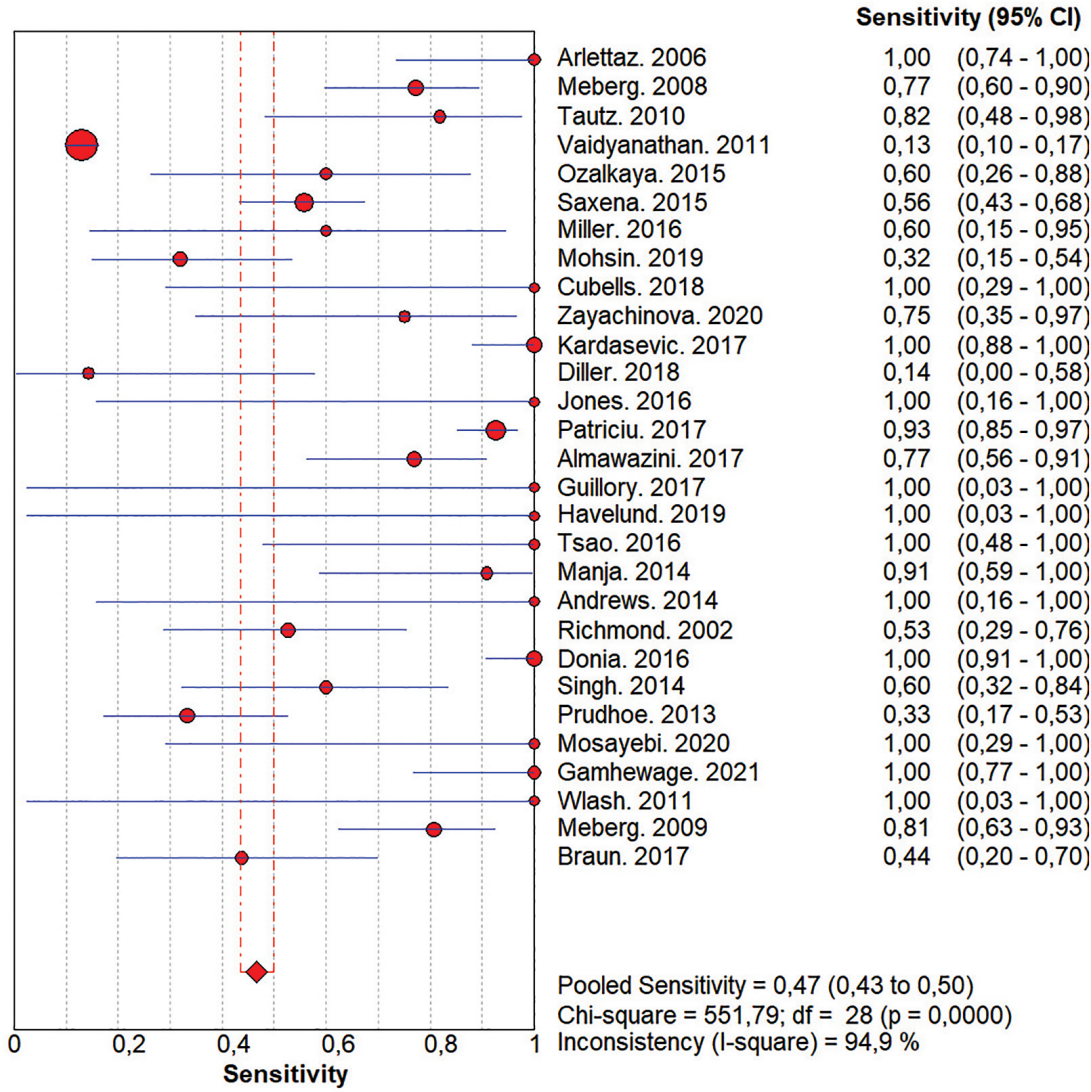
Forest graph demonstrating the results of the sensitivity meta-analysis. Curitiba, PR, Brazil, 2023. Source: The author (2023).

**Figure 3:**
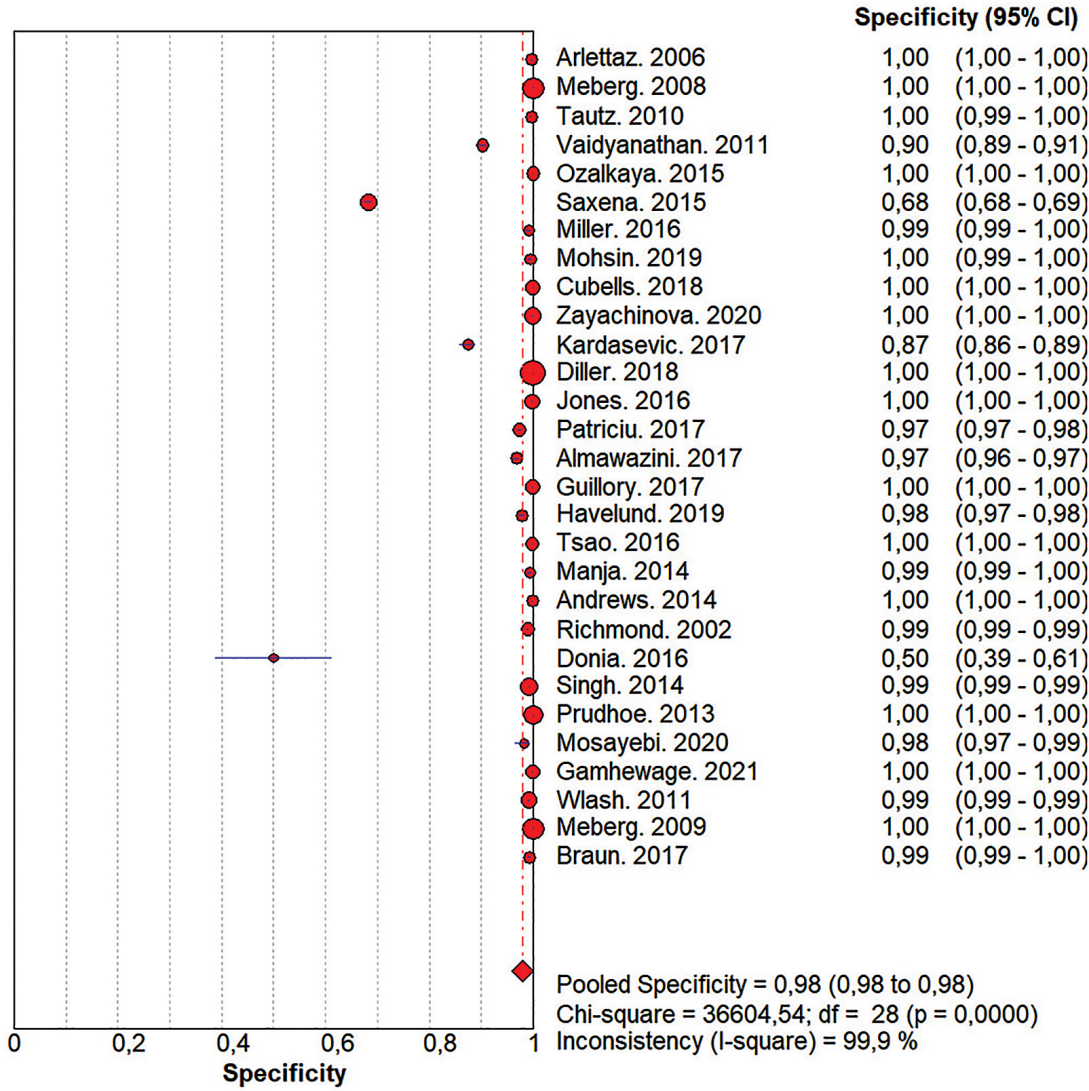
Forest plot demonstrating the results of the specificity meta-analysis. Curitiba, PR, Brazil, 2023. Source: The Author (2023).

False positive, false negative, true positive, true negative data were also collected, presented in Chart S2. First, a meta-analysis was carried out with 29^(20–48)^ studies and a total population of 388,491 newborns, which demonstrated a sensitivity of 47% (95% CI: 43% to 50%) (Figure 2) and a specificity of 98% (95% CI: 98% to 98%) (Figure 3). The high specificity of POT shows that the test is more suitable for identifying newborns without alterations.

Subsequently, subgroup analyses were carried out for sensitivity and specificity, according to the time of birth and the first POT measurement between 24 and 48 hours (Figures S1 and S2). In this meta-analysis, 20 studies were included^(21–24,27,28,31–33,35–38,40–42,44–48)^ with a sample of 203,992 participants. An increase in sensitivity to 67% (95% CI: 62% to 72%) and specificity to 97% (95% CI: 97% to 97) was demonstrated.

A third analysis including studies that were carried out with protocols that contained retests after the first measurement, comprising 13 studies^(21,24,27,28,31,32,34,35,37,38,42,44,45)^ and a sample of 124,469 participants was also developed (Figures S3 and S4). In this analysis, there was a similar increase in sensitivity to 91% (95% CI: 85% to 95%) and specificity to 100% (95% CI: 100% to 100%).

It was noticed that the studies differed regarding the retest interval. Meta-analysis was carried out with studies that carried out a retest after one hour of the first measurement, specifically with three studies^(24,36,45)^ and a sample of 16,802 participants (Figures S5 and S6). Sensitivity was 48% (95% CI: 31% to 66%) and specificity was 100% (95% CI: 100% to 100%).

Furthermore, there were studies that performed retests over a period of time greater than one hour and up to two hours, including eight studies^(21,23,28,31,32,35,37,41)^ and a total sample of 27,473 participants (Figures S7 and S8). In this case, sensitivity was 76% (95% CI: 65% to 84%) and specificity was 99% (95% CI: 99% to 99%).

An analysis was then carried out with studies that ­addressed the premature population, newborns with a gestational age of less than 36 weeks at birth. The analysis consisted of 4 studies^(23,39,45,46)^ and 124,469 studies (Figures S9 and S10). Through meta-analysis, specificity was 97% (95% CI: 96% to 97%) and sensitivity was 29% (95% CI: 25% to 33%).

After evaluating the certainty of the evidence using the GRADE system, according to the subgroup analyzed above, it was noticed that the *certainty of evidence remained moderate* for specificity regardless of sample characteristics. The reason for reducing the level of evidence was the high inconsistency highlighted by the meta-analysis. Regarding sensitivity, it was between very low, low and moderate, that is, the characteristics of the sample changed the level of evidence of the test sensitivity. Therefore, depending on the time of birth and application of the test, and the possibility of retesting, the heterogeneity of the sample was reduced and the level of evidence for POT sensitivity was raised.

Analysis of the methodological quality of the included studies demonstrated that all were classified as excellent quality, as they met the 10 evaluation criteria according to the JBI standardized checklist for diagnostic accuracy studies.

## DISCUSSION

The present systematic review demonstrated that POT has a specificity of 98% and sensitivity of 47%. Even though sensitivity has an important value for the accuracy of diagnostic tests, high specificity impacts on lower costs related to the lack of need for other tests^(49)^. Moreover, in the specific context of CHD, the high specificity of POT allows for the safe discharge of newborns with a negative test. Regarding the outcomes of interest in this review, the specificity rate is consistent with other studies^(43,44)^ published and the sensitivity result differs from previous studies, justified mainly by studies that used a saturation measurement protocol before 24 hours of birth, which presented lower values^(25,40,42,43,46)^.

Based on these results, a meta-analysis was carried out by subgroups. In the first subgroup, studies that carried out the test between 24 and 48 hours of the newborn’s life were selected. There was an increase in sensitivity to 67% and specificity remained high as indicated in the literature^(11)^. Screening carried out early, before 24 hours, presents higher false positive rates, due to the transition from fetal to neonatal circulation with an impact on pre- and post-ductal saturation^(50)^.

In the sample of this research, different protocols for POT were also identified. For example: screening was carried out without additional measurements, with (re)testing within 1 hour, within two, three and six hours of the first measurement. Among the studies that carried out the POT between 24h and 48h of birth, those that carried out a retest within one hour were also selected, and in another subgroup with a retest within two hours. We chose this classification because the divergent protocols are found in the literature^(6,11)^. It was noticed that the POT is dependent on the time of birth of the newborn, that is, the longer the time interval between measurements, the greater the sensitivity.

The study evidenced by Martin et al.^(11)^ justifies changing the POT protocol to a retest within one hour and, if a positive result, referral for an echocardiogram, based on the primary study by Diller et al.^(25)^, which has a sample of more than 77 thousand newborns. A simulation was carried out with the two protocols (one retest or two retests) and it was evident that the sensitivity was not changed, presenting a subtle increase in the false positive rate, which, as reported by the authors, is not a criterion for not adopting the protocol with a retest. It is worth highlighting that the study by Diller et al.^(25)^ (2018) presents POT sensitivity of 14% and was carried out before 24 hours of birth.

The change proposed by the Brazilian Society of Pediatrics presents the guidance to carry out two retests, after the first measurement, aiming to reduce the false positive rate. This protocol is presented by Kemper et al.^(51)^, the same study group as Martin et al.^(11)^. This study highlighted strategies for the safe and effective implementation of POT screening. Regarding the screening criteria, the group recommended the protocol with two retests. It should be noted that this study also suggests that each maternity hospital consider its particularities, demands of the newborn, family and health professionals. Furthermore, training professionals is essential for screening to be safe and effective.

With the reality of intensive care services, four studies were analyzed^(23,39,45,46)^, with a sensitivity of 21% and specificity of 97%, with a low and moderate level of evidence, respectively, which indicates that the POT for screening congenital heart diseases in this specific population is not effective. The authors note that it must be performed and interpreted in the context of other health care. The rate of false positives was higher in studies of intensive services than in studies with full-term populations, as pulmonary complications, prematurity, and other diseases impact the interpretation of saturation data^(52)^.

Only one study addressing screening in a home environment was included^(53)^. An effective test was configured for this context, which is especially important, given that the newborn is without clinical-hospital supervision. However, in this scenario, difficulties arise such as the cost of equipment, lack of professional training, and time required for retests, according to the protocol^(54)^. In this context, the possibility of portable oximeters, even with applications on cell phones, as a guide for performance and interpretation of results, would offer a reduction in costs and higher quality of screening^(55)^.

GRADE was chosen for each meta-analysis developed. Even with the grouping by subgroups of studies that methodologically showed similarities, inconsistency remained high. Guyatt et al.^(56)^ point out that inconsistency refers to the variability in the results and not in the characteristics of the studies, and high inconsistency does not demonstrate the incredibility of the results presented. Thus, there was no reduction in the level of evidence for this item by the authors, precisely due to the particularities of the diagnostic accuracy test of interest to the review developed. The imprecision item was responsible for reducing the level of evidence in this review, so that, among the subgroups, the level of evidence for the outcomes of this review was between moderate and high.

Suspected cases, those that require repeated measurement (90–94%), are impacted by the lack of an updated protocol, a trained team and the absence of a structured health network to assist these cases. Finally, it is worth highlighting that nurses provide immediate and mediate care to mother and child during the postpartum period. It has the indication and competence to carry out POT, qualifying assistance in neonatal screening. Thus, the status of knowledge of the correct parameters and possible changes to the test will guarantee better screening and coverage, highlighting, in this sense, that continuing education maintains its status of relevance for the quality of nursing care^(5,8,11)^.

Furthermore, the nurse is committed to informing and guiding those responsible for the newborn about neonatal screening, an action that will have a direct impact on its effectiveness. Furthermore, with this proximity to the clientele and guidance, the nurse promotes the reduction of family members’ anxiety regarding suspected or positive cases^(5,8,11,57)^.

Limitations include the lack of long-term follow-up of patients included in the studies and the inequality in the number of publications and specific protocols already established in developed and developing countries. It should also be highlighted that even with the divergences in the protocols presented, in all contexts evaluated, POT proved to be effective.

### Contributions to the Health Sector

The main strength of this systematic review consists of the sample of more than 300 thousand participants, highlighting the robustness of the data presented and that it is unlikely that the publication of new studies will have a significant impact on the sensitivity and specificity outcomes obtained. It is concluded, therefore, that the results presented demonstrate an advance in the knowledge and implementation of POT in practice as a viable, non-invasive test that can be widely used by nurses.

## CONCLUSION

This study allowed determining the accuracy of the Pulse Oximetry Test in screening for Congenital Heart Diseases in newborns in the first 48 hours of life. It is concluded that, for the early diagnosis of congenital heart diseases, the POT – Pulse Oximetry Test is a test of moderate sensitivity and high specificity, so that it can be said that it contributes to the diagnosis of negative cases. The profile of benefits presented by POT, and the low magnitude risks, favor its implementation.

According to a meta-analysis carried out in subgroups, POT is more effective when carried out within 24h – 48h of the birth of the newborn, and with protocols that present retests within two hours. It does not show satisfactory effectiveness for premature newborns. Regarding home births, no publication quantity was identified that would allow meta-analysis to be carried out. In the only study included, the test proved to be effective and safe, but limitations were highlighted in its implementation.

## Data Availability

The following online material is available from this article: https://doi.org/10.48331/scielodata.TVL6II

## Supplementary Material

The following online material is available for this article:

Chart S1 – Search strategies for the databases used – Curitiba, PR, Brazil, 2023.

Chart S2 – Data collection instrument according to identification of studies with false positive, false negative, true positive and true negative value – Curitiba, PR, Brazil, 2023.

Figure S1 – Forest graph demonstrating the results of the sensitivity meta-analysis of studies addressing POT between 24 and 48 hours of the newborn’s life – Curitiba, PR, Brazil, 2023.

Figure S2 – Forest graph demonstrating the results of the sensitivity meta-analysis of studies addressing POT between 24 and 48 hours of the newborn’s life – Curitiba, PR, Brazil, 2023.

Figure S3 – Forest graph demonstrating the results of the sensitivity meta-analysis of studies addressing POT with 1 or 2 retests – Curitiba, PR, Brazil, 2023.

Figure S4 – Forest graph demonstrating the results of the specificity meta-analysis of studies addressing POT top with 1 or 2 retests – Curitiba, PR, Brazil, 2023.

Figure S5 – Forest graph demonstrating the results of the sensitivity meta-analysis with retest in less than 2 hours – Curitiba, PR, Brazil, 2023.

Figure S6 – Forest graph demonstrating the results of the specificity meta-analysis with retesting at an interval of less than 2 hours – Curitiba, PR, Brazil, 2023.

Figure S7 – Forest graph demonstrating the results of the sensitivity meta-analysis with retesting at an interval greater than 2 hours – Curitiba, PR, Brazil, 2023.

Figure S8 – Forest graph demonstrating the results of the specificity meta-analysis of studies addressing POT top with 1 or 2 retests – Curitiba, PR, Brazil, 2023.

Figure S9 – Forest graph demonstrating the results of the meta-analysis of specificity of studies addressing premature newborns – Curitiba, PR, Brazil, 2023.

Figure S10 – Forest graph demonstrating the results of the sensitivity meta-analysis of studies addressing premature newborns – Curitiba, PR, Brazil, 2023.
