# Supplementary material for: Pulse oximetry test for screening congenital heart diseases: a systematic review
Source: Rev Esc Enferm USP. 2024 Mar 1;57:e20230215. doi: 10.1590/1980-220X-REEUSP-2023-0215en (PMC10906467; doi:10.1590/1980-220X-REEUSP-2023-0215en)
Supplement: Supplementary file 1 [file 1980-220X-reeusp-57-e20230215-suppl1.pdf]

Rev Esc Enferm USP
https://doi.org/10.1590/1980-220X-REEUSP-2023-0215en

Supplementary Material to "Pulse oximetry test for screening congenital heart diseases: a systematic review"

Chart S1 – Search strategies for the databases used – Curitiba, PR, Brazil, 2023.

**DATABASE**	**STRATEGY**	**Number of studies identified**
PUBMED	((((((((((infant, newborn[MeSH Terms]) OR (Newborn[Title/Abstract])) OR (Neonate[Title/Abstract])) OR (Infant, Postmature[MeSH Terms])) OR (Infant, Premature[MeSH Terms])) OR (Infant, Preterm[MeSH Terms])) OR (infant, newborn [MeSH Terms])) OR (Newborn [Title/Abstract])) OR (Neonate [Title/Abstract])) OR (infant, postmature [MeSH Terms])) OR (infant, premature [MeSH Terms])) OR (infant, premature [MeSH Terms]) AND (((((Oximetry[MeSH Terms]) OR (Pulse oximetry[Title/Abstract])) OR (Pulse oximetry screening[Title/Abstract])) OR (oximetry [MeSH Terms])) OR (pulse oximetry [Title/Abstract])) OR (pulse oximetry screening [Title/Abstract]) AND ((((((((Heart Defects, Congenital[MeSH Terms]) OR (Abnormality, Heart[Title/Abstract])) OR (Congenital Heart Defect[Title/Abstract])) OR (Malformation Of Heart[Title/Abstract]))	489

	OR (Congenital Heart Disease[Title/Abstract])) OR (heart defects, congenital [MeSH Terms])) OR (abnormality heart [Title/Abstract])) OR (congenital heart defect [Title/Abstract])) OR (malformation of heart [Title/Abstract])) OR (congenital heart disease [Title/Abstract])	
CINAHL	TX infant, newborn OR TX newborn OR TX (neonate or neonatal or premature or preterm or newborn or infant) AND TX oximetry OR TX pulse oximetry OR TX pulse oximetry screening AND TX heart defects, congenital OR TX abnormality, heart OR TX congenital heart defects OR TX malformation of heart OR TX congenital heart disease OR TX malformation	276
Embase	('newborn'/exp OR 'child, newborn' OR 'full term infant' OR 'human neonate' OR 'human newborn' OR 'infant, newborn' OR 'neonate' OR 'newborn' OR 'newborn baby' OR 'newborn child' OR 'newborn infant' OR 'newly born baby' OR 'newly born child' OR 'newly born infant' OR 'prematurity'/exp OR 'extremely premature infant' OR 'infant, extremely premature' OR 'infant, premature' OR 'infant, premature, diseases' OR 'neonate, premature' OR 'pre-mature infant' OR 'pre-term baby' OR 'pre-term child' OR 'pre-term infant' OR 'pre-term neonate' OR 'pre-term newborn' OR 'premature' OR 'premature baby' OR 'premature birth' OR	604

	'premature child' OR 'premature childbirth' OR 'premature infant' OR 'premature infant disease' OR 'premature infant diseases' OR 'premature neonate' OR 'premature newborn' OR 'premature syndrome' OR 'prematuritas' OR 'prematurity' OR 'preterm baby' OR 'preterm child' OR 'preterm infant' OR 'preterm neonate' OR 'preterm newborn' OR 'postmaturity'/exp OR 'infant, postmature' OR 'post maturity' OR 'post-mature baby' OR 'post-mature infant' OR 'post-mature neonate' OR 'post-term infant' OR 'post-term newborn' OR 'postmature baby' OR 'postmature child' OR 'postmature infant' OR 'postmature neonate' OR 'postmature newborn' OR 'postmaturity' OR 'postterm infant') AND ('congenital heart malformation'/exp OR 'congenital heart anomaly' OR 'congenital heart defect' OR 'congenital heart defects' OR 'congenital heart malformation' OR 'heart anomaly' OR 'heart congenital anomaly' OR 'heart congenital defect' OR 'heart congenital malformation' OR 'heart defects, congenital' OR 'heart malformation') AND ('oximetry'/exp OR 'blood oxygen measurement' OR 'oximetry' OR 'oxygen measurement, blood' OR 'oxymetry' OR 'pulse oximetry'/exp OR 'oximetry, pulse' OR 'pulse oximetry' OR 'pulse oximetry screening')	

SCOPUS	TITLE-ABS-KEY(infant, newborn OR newborn OR neonate OR infant, postmature OR infant, premature OR infant, preterm) AND TITLE-ABS-KEY(heart defects, congenital OR abnormality, heart OR congenital heart defect OR malformation of heart OR congenital heart disease) AND TITLE-ABS-KEY(oximetry OR pulse oximetry OR pulse oximetry screening)	10
WOS	TS=(infant, newborn OR newborn OR neonate OR infant, postmature OR infant, premature OR infant, preterm) AND TS=(heart defects, congenital OR abnormality, heart OR congenital heart defect OR malformation of heart OR congenital heart disease) AND TS=(oximetry OR pulse oximetry OR pulse oximetry screening)	400
CAPES	oximetria de pulso AND cardiopatias congênitas	19
OATD	pulse oximetry AND congenital heart desease	16
WWSO	pulse oximetry AND congenital heart desease and newbor	59
