# Supplementary material for: Pulse oximetry test for screening congenital heart diseases: a systematic review
Source: Rev Esc Enferm USP. 2024 Mar 1;57:e20230215. doi: 10.1590/1980-220X-REEUSP-2023-0215en (PMC10906467; doi:10.1590/1980-220X-REEUSP-2023-0215en)
Supplement: Supplementary file 2 [file 1980-220X-reeusp-57-e20230215-suppl2.pdf]

Rev Esc Enferm USP
https://doi.org/10.1590/1980-220X-REEUSP-2023-0215en

Supplementary Material to "Pulse oximetry test for screening congenital heart diseases: a systematic review"

Chart S2 - Data collection instrument according to identification of studies with false positive, false negative, true positive and true negative value – Curitiba, PR, Brazil, 2023.

ID	False Positive	False Negative	True Positive	True Negative
1	94	6	20	2841
2	1	0	2	1905
3	7	0	12	3238
4	25	9	7	3382
5	2	0	3	8851
6	33	6	1	77114
7	41	0	38	41
8	5	0	14	8699
9	10	0	1	11311
10	58	0	1	2737
11	21	0	2	10237
12	215	0	29	1501
13	8	1	10	1426
14	3	8	27	48648
15	0	6	25	57878
16	13	2	3	1582
17	8	17	8	1617
18	7	0	3	403
19	1	4	6	8197

20	138	7	87	5174
21	0	20	10	29900
22	54	9	10	5553
23	6009	31	39	12930
24	199	6	9	25645
25	9	2	9	3316
26	11	0	5	6280
27	494	370	55	4568
28	112	0	1	14451
29	12	2	6	20527

Legend: ID – Identification of the article.
