# Supplementary material for: Pulse oximetry test for screening congenital heart diseases: a systematic review
Source: Rev Esc Enferm USP. 2024 Mar 1;57:e20230215. doi: 10.1590/1980-220X-REEUSP-2023-0215en (PMC10906467; doi:10.1590/1980-220X-REEUSP-2023-0215en)
Supplement: Supplementary file 11 [file 1980-220X-reeusp-57-e20230215-suppl11.pdf]

Rev Esc Enferm USP

https://doi.org/10.1590/1980-220X-REEUSP-2023-0215en

Supplementary Material to “Pulse oximetry test for screening congenital heart diseases: a systematic review”

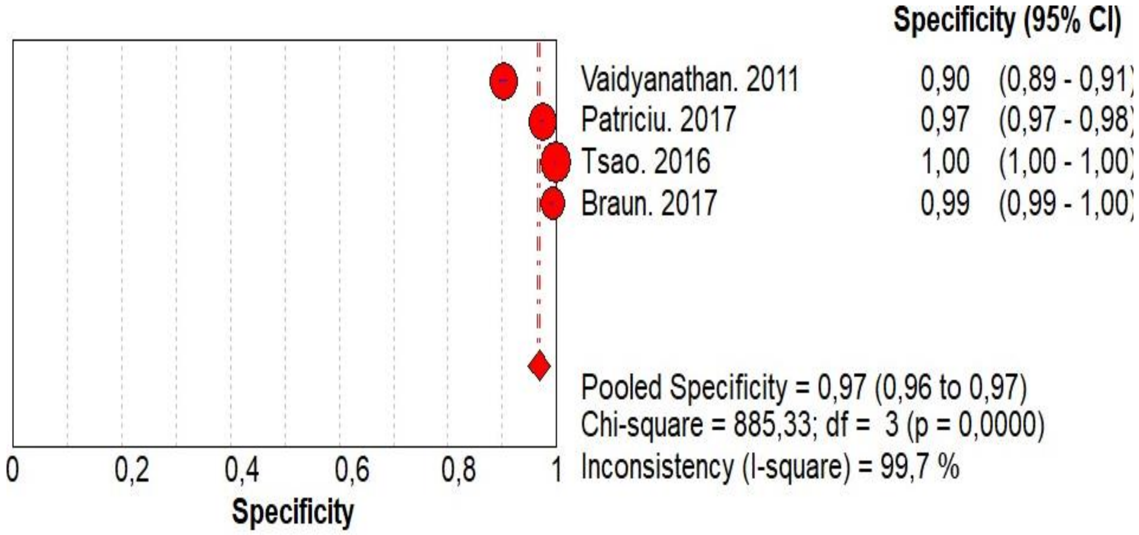

Figure S9 – Forest graph demonstrating the results of the meta-analysis of specificity of studies addressing premature newborns – Curitiba, PR, Brazil, 2023.
